# Commentary: The Emerging Neuroscience of Third-Party Punishment

**DOI:** 10.3389/fnhum.2017.00512

**Published:** 2017-10-24

**Authors:** Oksana Zinchenko, Vasily Klucharev

**Affiliations:** ^1^Centre for Cognition and Decision Making, National Research University Higher School of Economics, Moscow, Russia; ^2^Department of Psychology, National Research University Higher School of Economics, Moscow, Russia

**Keywords:** third-party punishment, default mode network, central-executive network, transcranial direct current stimulation, temporoparietal junction, dorsolateral prefrontal cortex, functional connectivity, social norms

More than a decade of neuroimaging research has established that several distinct brain networks are consistently recruited during *social punishment*, i.e., the propensity of cooperative individuals to spend some of their resources penalizing norm violators. Studies in behavioral economics have shown that social punishment can explain why genetically unrelated individuals are often able to maintain high levels of socially beneficial cooperation (Fehr and Gächter, 2002; de Quervain et al., 2004; Gureck et al., 2006). In particular, social norms can be reinforced by parties that are directly affected by norm violators (“*second parties*” punishment—SPP) and parties that are financially unaffected (“*third parties*” —TPP) (Fehr and Fischbacher, 2004). Importantly, norm violations often do not hurt other people directly. Thus, third-party sanctions are particularly effective at reinforcing group norms that regulate human behavior (Bendor and Swistak, 2001; Fehr and Fischbacher, 2004).

Pioneering behavioral studies have showed that strong emotions trigger the willingness to punish norm violators (Hirshleifer, 1987; Frank, 1988; Fehr and Gächter, 2002); in particular, TPP is motivated by both empathy toward the victim and anger toward the norm violator (Batson et al., 2007; Pedersen, 2012). Recently, neuroimaging studies have demonstrated a critical role of *executive* (the dorsolateral prefrontal cortex, DLPFC) and *mentalizing* (the temporoparietal junction, TPJ) brain regions in TPP (Baumgartner et al., 2012; Bellucci et al., 2016). Thus, neuroscience studies could help to further develop psychological theories of TPP by clarifying the specific neurocognitive mechanisms triggering punishment decisions in various social contexts.

Recently, Krueger and Hoffman (2016) reviewed and summarized the roles of three brain networks that are activated during TPP: the salience network (SN), the default mode network (DMN), and the central executive network (CEN). First, they suggested that the SN (the insula, amygdala, and dorsal anterior cingulate) detects and generates an aversive experience that initiates TPP. Second, the authors argued that the DMN (the medial prefrontal cortex, posterior cingulate cortex, and TPJ) integrates the perceived harm and inference of intentions into an assessment of blame. Finally, they proposed that the CEN (the dorsolateral prefrontal cortex and posterior parietal cortex) converts the blame signal into a specific punishment decision.

Interestingly, these three networks partially overlap with those underlying the detection of norm violations in other social contexts. There is a growing cognitive neuroscience literature on a neural mechanism that detects when individual behavior or beliefs differ from those of others (for reviews, see Izuma, 2013; Klucharev and Shestakova, 2015). A number of neuroimaging studies have demonstrated that the activity of the SN, DMN, and CEN encodes perceived deviations from group norms (Klucharev et al., 2009; Berns et al., 2010; Campbell-Meiklejohn et al., 2010; Izuma and Adolphs, 2013). In particular, the insula, dorsal anterior cingulate, medial prefrontal cortex, posterior cingulate cortex, and DLPFC have all been implicated in norm monitoring. Interestingly, many of these studies also reported norm-monitoring activity in the ventral striatum (Klucharev et al., 2009; Crockett et al., 2013; Xiang et al., 2013), which is a key region implicated in reward valuation. Despite the fact that the ventral striatum was not mentioned by Krueger and Hoffman (2016), recent studies have also implicated this region in TPP (Strobel et al., 2011; Hu et al., 2015), which further indicates that these two lines of research (detection of norm violations and TPP) share common neural mechanisms and should be further integrated.

However, amygdala activity was reported only in SPP and TPP studies (Buckholtz et al., 2008; Yu et al., 2015; Ginther et al., 2016). This can be explained by the financial losses and harms associated with this paradigm. TPJ activity also seems to be specific to the context of TPP (Baumgartner et al., 2012, 2014). A recent quantitative review suggested that the TPJ consists of functionally and spatially distinct neuroanatomical sub-regions specializing in different cognitive processes (Schurz et al., 2017). It has been hypothesized that the TPJ supports the processing of social contexts that require the representation of (a) the social context (stimuli) and (b) the context provided by attention, memory, and language (Carter and Huettel, 2013). These convergent processes constitute a theory of mind. This ability to make inferences about other people's mental states, which is associated with the TPJ, is critical to the ability to blame them for violations of complex context-dependent social norms. Thus, to uncover the neural mechanisms of TPP, it is essential to clarify the neurocomputational mechanism that allows the TPJ (as a part of the DMN) to link norm-violation detection (SN) to specific punishments (CEN).

Interestingly, TPJ activity during TPP is paralleled by an initial deactivation of the DLPFC (Buckholtz et al., 2008). This indicates functionally opposed neural activity in these two regions. The DLPFC demonstrates a biphasic neural activity—following initial deactivation, it increases activity—when subjects make the final decision to punish “based on assessed responsibility and blameworthiness” (Buckholtz et al., 2008, p. 935). Thus, it is important to explain the “antagonistic” relationship between the DMN (TPJ) and CEN (DLPFC). Many recent studies have evaluated functional and effective connectivity during SPP (Yu et al., 2015) and TPP (Treadway et al., 2014; Bellucci et al., 2016). They demonstrated that the lateral regions of the prefrontal cortex receive an input from the TPJ during SPP (Yu et al., 2015), while the dorsomedial prefrontal cortex plays the role of a hub, coordinating DLPFC and TPJ activity during the decision stage of TPP (Bellucci et al., 2016). Neuroimaging studies have demonstrated that the temporoparietal-medial-prefrontal circuit suppresses the amygdala during evaluations of unintentional harm (Treadway et al., 2014; Yu et al., 2015) in both SPP and TPP or boosts amygdala activity and strengthens its connectivity with the lateral prefrontal regions (during TPP) when a harm is intentional (Treadway et al., 2014). This suggests that the temporoparietal-medial-prefrontal circuit gates the emotional responses to norm violations and regulates subsequent reactive punishment.

These recent findings raise intriguing and testable questions for future research, e.g., in the use non-invasive brain stimulation to further verify fMRI findings. There is evidence suggesting that transcranial current stimulation could effectively modulate within- and between-network interactions. For example, transcranial alternating current stimulation induced oscillatory desynchronization between the medial frontal and parietal cortices and, therefore, affected value-based decisions but not closely matched perceptual decisions (Polanía et al., 2015). Simultaneous anodal transcranial direct current stimulation of the DLPFC, together with cathodal stimulation of the supraorbital region, led to changes in the default mode network and frontal-parietal networks (Keeser et al., 2011) and increased synchrony within the focused attention network (Peña-Gómez et al., 2012). According to Buckholtz et al. (2008), the CEN exerts an inhibitory influence over the DMN in order to program decisions about an appropriate punishment. Thus, a person could use a simultaneous application of transcranial direct or alternating current stimulation to the TPJ and DLPFC in order to modulate an antagonistic CEN/DMN interaction during TPP. We speculate that an enhancement of TPJ activity, along with the simultaneous suppression of DLPFC activity, should enhance an antagonistic CEN/DMN interaction and lead to increased TPP. The aforementioned behavioral effect should be associated with changes in the functional connectivity between the TPJ and DLPFC. A combined non-invasive brain stimulation-neuroimaging approach could further uncover the complex intrinsic network dynamics in the brain, which underlies TPP.

## Author contributions

All authors listed have made substantial, direct and intellectual contribution to the work, and approved it for publication.

### Conflict of interest statement

The authors declare that the research was conducted in the absence of any commercial or financial relationships that could be construed as a potential conflict of interest.
